# The aqueous supramolecular chemistry of crown ethers

**DOI:** 10.3389/fchem.2023.1119240

**Published:** 2023-01-20

**Authors:** Zhenhui Qi, Yao Qin, Jijun Wang, Maojin Zhao, Zhuo Yu, Qiangqiang Xu, Hongqi Nie, Qilong Yan, Yan Ge

**Affiliations:** ^1^ Sino-German Joint Research Lab for Space Biomaterials and Translational Technology, Synergetic Innovation Center of Biological Optoelectronics and Healthcare Engineering (BOHE), Shaanxi Provincial Synergistic Innovation Center for Flexible Electronics & Health Sciences (FEHS), School of Life Sciences, Northwestern Polytechnical University, Xi’an, Shaanxi, China; ^2^ Science and Technology on Combustion, Internal Flow and Thermostructure Laboratory, Northwestern Polytechnical University, Xi’an, China

**Keywords:** crown ethers, selenium, responsive materials, hofmeister series, structural water

## Abstract

This mini-review summarizes the seminal exploration of aqueous supramolecular chemistry of crown ether macrocycles. In history, most research of crown ethers were focusing on their supramolecular chemistry in organic phase or in gas phase. In sharp contrast, the recent research evidently reveal that crown ethers are very suitable for studying abroad range of the properties and applications of water interactions, from: high water-solubility, control of Hofmeister series, “structural water”, and supramolecular adhesives. Key studies revealing more details about the properties of water and aqueous solutions are highlighted.

## Introduction

Supramolecular chemistry provides a powerful platform for achieving complex chemical/biological activities using non-covalent interactions. Water is significant for biomimetic chemistry and to achieve sustainability in both the natural environment and human societies. Thus, exploring aqueous supramolecular chemistry is essential for producing advanced functional materials for biomedical processing, energy, information technology, and environmental science applications (Zayed et al., 2010; Cremer et al., 2017; Zheng et al., 2018; Gatiatulin et al., 2019; Xiao et al., 2020a; Duan et al., 2020; WuXiao, 2020; Zhang et al., 2020; Zhang et al., 2021a; Zhang et al., 2021b; Ding et al., 2021; Liu et al., 2021; Zhu et al., 2021; Shi et al., 2022).

Crown ethers (CEs) are cyclic compounds comprising several ether linkages with a specifically sized cavity in their centers. The discovery of CEs was pivotal in the establishment of supramolecular chemistry. CE supramolecular chemistry is predominantly conducted in organic solvents (Qi et al., 2012; Qi et al., 2014; Qi and Schalley, 2014; Ali et al., 2019; Ge et al., 2019; Xiao et al., 2021) or partially in the gas phase (Weimann et al., 2009; Qi et al., 2013). The molecular structure of CEs indicates their great flexibility to adapt their conformation to interact with water molecules. In organic media, the lone electron pairs on the CE oxygen atoms create a region of high electron density in the ring cavity. CEs exhibit a rich conformational panorama and low energy barriers, and research in the past two decades has focused on fabricating CE-based threads or interlocking components for use in nanomachines. These reactions have primarily been conducted in organic solvents; however, in aqueous media, the polar surfaces of CE molecules are exposed, with a water-accessible surface area (ASA) originating from the ethylene glycol units. Therefore, it is reasonable to expect that CEs can be water-soluble macrocycles. However, the scientific community has long been skeptical of the solubility of CEs in water, resulting in few reported studies in the literature of water-soluble CEs (Zhang et al., 2022a; Wang et al., 2022) compared to those on other macrocycles, such as cucurbiturils (Das et al., 2019), calixarenes (Xu et al., 2019), cyclodextrins (Kwong et al., 2021; Li et al., 2021; Luo et al., 2021; Zhang et al., 2022b), and pillararenes (Yu et al., 2021). Recently, CEs have emerged as an intriguing host for studying water and aqueous supramolecular chemistry.

In this mini-review, we summarize applications that have capitalized on CE–water interactions to fabricate aqueous materials. Unexpectedly high water solubility, control of the Hofmeister cationic series, and “structural water” in supramolecular adhesives are highlighted.

## CEs with water: A missing type of water-soluble macrocycle

**FIGURE 1:**
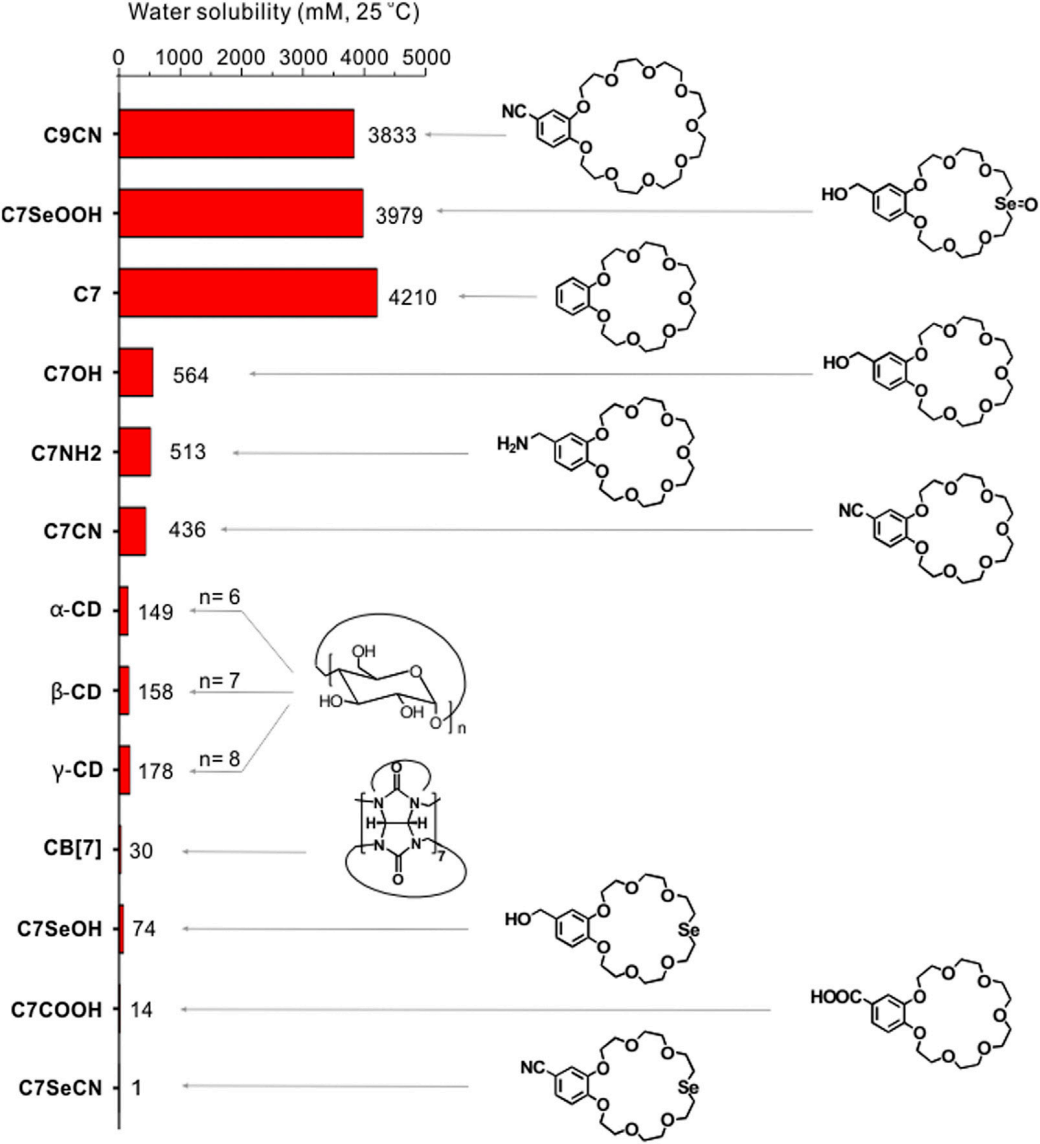
Comparison of the water-solubility of CEs and their derivatives with *α*-cyclodextrin, *β*-cyclodextrin, and *γ*-cyclodextrin and cucurbit[7]urils. Adapted and reproduced from Dong et al. (2017a), Jin et al. (2019), Xu et al. (2021) with kind permission from American Chemical Society and Elsevier.

In 2017, Qi et al. serendipitously discovered that benzo-21-crown-7 ether (**C7**) exhibits remarkably high water solubility (Dong et al., 2017a). The solubility of **C7** in water at room temperature reaches 1,500 g/L (4.21 M), which is superior to those of many classic water-soluble supramolecular macrocycles, such as cucurbit [n]urils and *α*-, *β*-, and *γ*-cyclodextrins (see comparison in Figure 1). Concentration-dependent nuclear Overhauser effect spectra (NOESY) revealed no significant intermolecular interactions between the **C7** molecules in solution. Diffusion-ordered NMR spectra (DOSY) indicated that the **C7** diffusion constants at high and low concentrations were similar. The solution was prepared using the **C7** monomer and no molecular aggregation was observed. The functional group on the benzo-moiety significantly affects the water-solubility of **C7** (Figure 1). The solubility of the derivatives follows the sequence: **C7** > **C7NH2** > **C7CN** > **C7COOH**. Entropic desolvation of the CE ethylene glycol units is known to increase the hydrophobicity of the cyclic chain, which results in separation from the aqueous solution. Therefore, the thermo-responsivity of **C7** and its derivatives in water is an intriguing topic of study (Zhang et al., 2021c). For example, low-molar-mass **C7** and **C7CN** exhibit lower critical solution temperature (LCST) phenomena. Interestingly, **C7COOH** exhibits upper critical solution temperature (UCST) behavior followed by LCST phase behavior, implying that the benzo-group functionalization is instrumental in modulating the thermo-responsiveness of the CEs.

**FIGURE 2:**
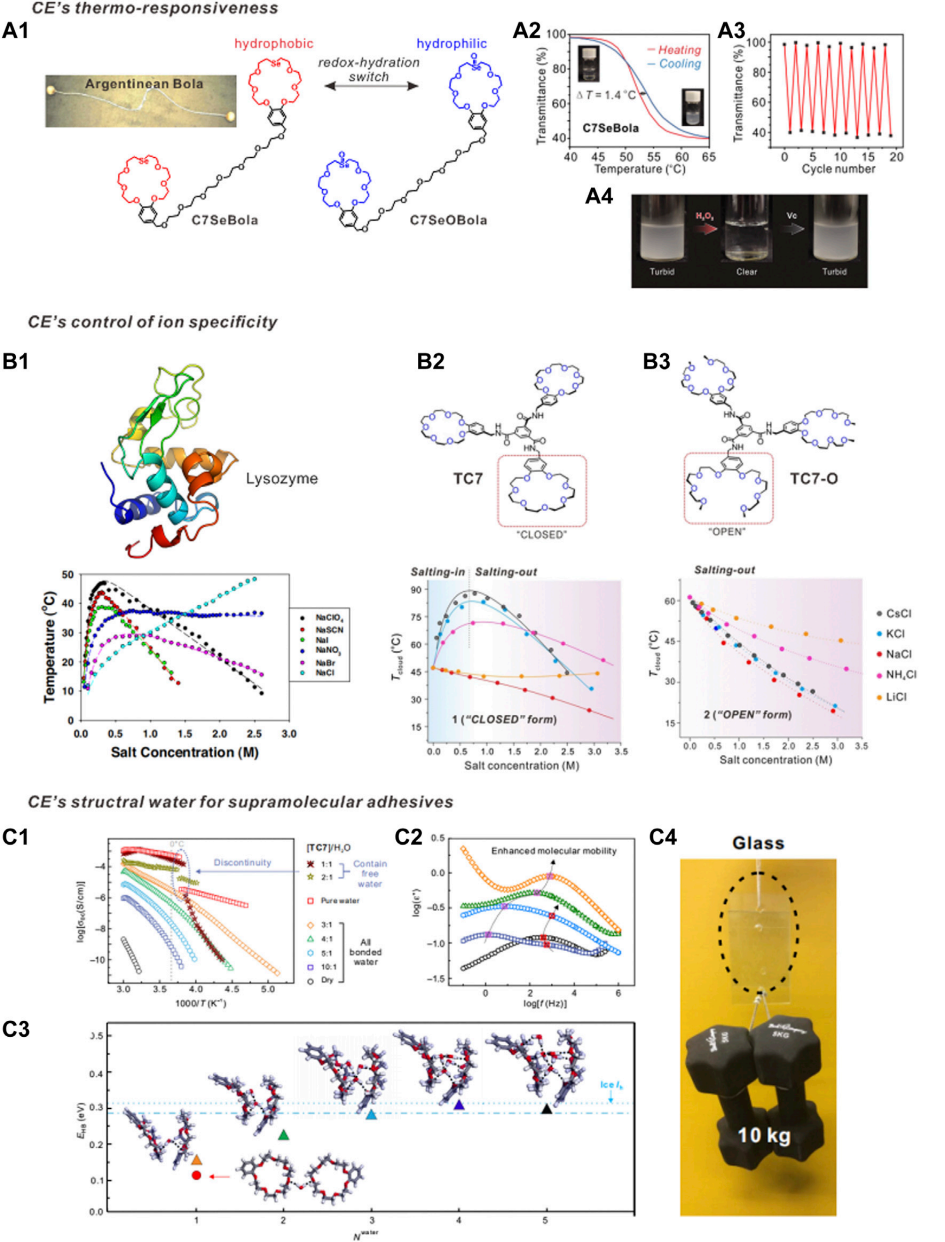
**(A)** The redox-controlled hydration of CE for design macrocyclic amphiphiles. **(A1)** the chemical structure of bolaamphiphile **C7SeBola**; **(A2) C7SeBola** exhibited rapid LCST behavior in water, **(A3)** The highly reversible LCST transition processes, **(A4)** turbid-to-clear and clear-to-turbid transitions can be controlled in an “ON-OFF” manner. **(B)** Hofmeister series reversal behavior. **(B1)** Hofmeister series reversal of anions for lysozyme reported by Cremer et al. The apparent association constants of anionic-lysozyme interaction for ClO_4_^−^, SCN^−^, I^−^, NO_3_^−^, Br^−^ and Cl^−^ are 0.10, 0.10, 0.09, 0.10, 0.18 and 0.12 M^−1^, respectively. **(B2)** Hofmeister series reversal of cation for a triple-CE-molecule (**TC7**). The inverse Hofmeister series at low salt concentration regimes should be the cation–solute interaction at wok. The charge density is initially dominant and then decays due to the increase of the ionic radius, then the enhanced “size-fitting” induced molecular recognition dominants the system. **(B3)** Ring-open counterpart (**TC7-O**) showed no Hofmeister series reversal. **(C) TC7** generates structural water for fabricating supramolecular adhesive material. **(C1)** The dependence of DC conductivity $\sigma_{dc}$ versus 1/T for **TC7**_n_-**H** materials with different water content. **(C2)** Dielectric loss versus frequency for **TC7**_n_-**H** materials with different water content at a temperature of −100°C; **(C3)** Averaged hydrogen-bond strengths (EHB) of CE-water systems with different number of water molecules (*N*_water_) as obtained from the density functional theory (DFT) calculations; **(C4)** Application of **TC7**_10_-**H**_1_ materials as supramolecular adhesive materials. Adapted and reproduced from Xu et al. (2021), Qi et al. (2018), Zhang and Cremer (2009), Dong et al. (2017b) with kind permission by the Elsevier, National Academy of Science, Wiley-VCH and American Association for the Advancement of Science.

Substituting the O atom opposite the benzo-moiety on the CE with an Se atom transformed the **C7CN** from hydrophilic to strongly hydrophobic, and the aqueous solubility of **C7SeCN** decreased to 1 mM (Figure 1) (Jin et al., 2019). Using detailed computer modeling, the solvation free energies ( ${∆G}_{s}^{\theta }$ ) of **C7CN** and **C7SeCN** were calculated to be −104.21 and −81.82 kJ/mol, respectively, which are in accordance with the experimentally determined solubility trend. By contrast, when the substituent on **C7CN** is a selenoxide moiety, the water-solubility of the CE is significantly enhanced. Both the crystal structure and theoretical modelling revealed that selenoxides are excellent hydrogen-bond (HB) acceptors, because the HB length of the selenoxide group (1.72 Å) is significantly shorter than that of the ether (-O-) group (1.90 Å). Qi et al. found that the water solubility of **C7SeOOH** reaches 3,979 mM (Xu et al., 2021). By contrast, the water solubility of **C7OH** is 564 mM and that of **C7SeOH** is merely 74 mM. Based on these results, the reversible transformation between the selenide- and selenoxide-containing CEs creates a switchable macrocycle pair (Figure 2A1) that differs dramatically in hydration and is controlled by redox stimuli. This constitutionally adaptive behavior of CEs offers an elegant platform for controlling and/or switching the hydration of bolaamphiphile skeletons (which are similar in structure to Argentinean boleadoras) (Xu et al., 2021). The resulting macrocyclic bolaamphiphile **C7SeBola** exhibited LCST behavior in water (Figure 2A2), and the overall LCST transition processes were highly reversible (Figure 2A3). Based on the redox-responsive transformation between **C7SeBola** and **C7SeOBola**, the chemically responsive, reversible, turbid-to-clear and clear-to-turbid transitions can be controlled in an “ON-OFF” manner (Figure 2A4). Based on these results, diverse CE-based macrocyclic amphiphiles with unique structures and responsiveness were subsequently developed (Zheng et al., 2019; Li et al., 2020a; Deng et al., 2020; Wu et al., 2020).

## CEs with ions: Revisiting the Hofmeister series

The chemistry of aqueous salt solutions is rich in ambiguities and a prime example is the Hofmeister effect, which is a prominent example of a multilateral interactive relationship (Jungwirth and Cremer, 2014). As early as 1888, Franz Hofmeister, a Czech protein scientist, noticed specific cationic and anionic effects on the solubility of proteins and discovered that the effectiveness of simple ions in protein precipitation followed a specific order (the Hofmeister series). The effectiveness of the influence of positive ions on precipitating proteins is smaller than negative charged ions (Aoki et al., 2016). Subsequently, a wide variety of phenomena, from protein folding and enzymatic activity to colloidal assembly and bacterial growth, have been shown to follow the Hofmeister series (Wang et al., 2021). Recent evidence suggests that Hofmeister effects arise from direct interactions between ions and solutes and between ions and the first solvation shells of those solutes. However, several observations have challenged these theoretical ideas, such as the Hofmeister series reversal. The reversal of the Hofmeister series between low and high salt concentration regimes was initially reported in 1911. However, explanation and prediction of such phenomena are not possible using existing theories on specific ion effects. The current prevailing theory, the law of matching water affinities (LMWA), can only qualitatively explain and rank ion–ion and ion–solute interactions on charged surfaces. The rules and parameters that govern the reversal phenomena on a neutral polar surface, such as the uncharged hydrophilic regions on proteins or synthetic materials, have not yet been determined.

To address this issue, Qi and Dong developed a low-molecular-weight supramolecular system to reveal the underlying systematic relationships between ions, water, and solutes (Qi et al., 2018). A triple-CE-molecule (**TC7**) and its ring-opened counterpart (**TC7-O**) exhibit a distinctive topological effect (Figures 2B2, B3). When neutral polar groups are arranged in a crown geometry, the “size-fitting”-induced solute-cation recognition, though rather weak, efficiently tunes the Hofmeister series and results in a Hofmeister series reversal phenomenon with singly charged cations. The phase transition curve of **TC7** with cations (Figure 2B2, bottom) is quite similar to that of lysozyme with anions (Figure 2B1, bottom) reported by Cremer et al. (Zhang and Cremer, 2009). Interestingly, isothermal titration calorimetry measurements revealed unexpectedly low cation-solute interactions in this Hofmeister series reversal, with an apparent equilibrium association constant *K*a of ∼10 M^−1^. Similar low binding affinities, from a supramolecular chemistry viewpoint, are observed in the majority of reported supramolecular recognition motifs (*K*a > 10^3^ M^−1^). Therefore, CEs provide an elegant and simple model to control the Hofmeister series reversal behavior, which has long been a topic of debate in the physics community (Jungwirth and Cremer, 2014).

On-going research has further demonstrated the general principles of the spatial topology factor and site-fitting effect in CE-containing ionic systems (Huang et al., 2018; Luo et al., 2019; Pan et al., 2021). Chen et al. observed a similar coexisting salting-in and salting-out effect (Huang et al., 2018) when **C7** was functionalized on a poly (vinyl alcohol) polymer system, demonstrating that CEs can serve as general building blocks to control ion specificity in aqueous media. A benzo-27-crown-9 ether (**C9OH**) system was used to explore the “size-fitting”-induced host-guest complexation between **C9** and guanidinium. A similar coexisting salting-in and salting-out effect can be extended to guanidinium-type ions, such as guanidinium chloride and arginine (Pan et al., 2021).

## CEs with structural water: Water act as an essential comonomer in supramolecular polymerization

Structural water molecules form strong HBs with the polar groups in proteins and are thought to tighten the protein matrix. These deeply-buried water molecules are considered an integral part of the folded protein structure. The concept of structural water bound within hydrophobic capsules that stabilize protein structures is well established in biology; however, the stabilization effects of water in materials science are rarely discussed. Supramolecular polymers are formed through non-covalent, directional interactions between monomeric building blocks. Assembly of these polymers is reversible, which enables functions such as coating, self-healing, and responsiveness (Wei et al., 2014; Xiao et al., 2020b; Lv et al., 2021). Water is typically regarded as a solvent in the assembly of supramolecular polymers; however, in 2017, Qi and Dong et al. reported a supramolecular polymer in which water acted as an indispensable comonomer in the supramolecular polymerization (Dong et al., 2017b).

In the dry state, **TC7** is a fragile, slippery solid that has an amorphous glassy structure. However, when exposed to the ambient environment (25°C, 40% relative humidity), the dry **TC7** quickly adsorbed the ambient water vapor, affording a **TC7**-water mixture with notable flexibility, elasticity, and adhesive properties (Figure 2C). Infrared spectra indicated that the **TC7** molecules self-assembled into one-dimensional supramolecular aggregates *via* amido-HBs. Broadband dielectric spectra (BDS) provided key evidence of the existence of structural water molecules (Figures 2C1, C2). The frequency-dependence of the conductivity spectra of **TC7**_n_-water(**H**)_1_ (*n* = 1, 2, 3, 4, 5, 10; *n* denotes the weight ratio of **TC7** to added water) presented the typical conductivity-frequency behavior observed in semiconducting materials (Zhang et al., 2021c). For weight ratios of **TC7**_n_-**H**_1_ above *n* > 2 (Figure 2C1), the $\sigma_{dc}$ versus 1/T plots did not exhibit any discontinuity at the freezing temperature of water (273 K), indicating that up to this weight ratio, water molecules in this supramolecular polymer exist as structural water (which is distinct from free water in the bulk solvent). By contrast, the $\sigma_{dc}$ versus 1/T plots of both **TC7**_2_-**H**_1_ and **TC7**_1_-**H**_1_ displayed a clear discontinuity close to the temperature at which water in the bulk solvent also displays a discontinuity. This result clearly indicates the presence of significant amounts of free water molecules within this supramolecular material that freeze at the temperature at which the discontinuity is observed. An *in situ* single crystal structure of frozen water was obtained from the high-water content sample **TC7**_1_-**H**_1_, which confirmed these findings. The average hydrogen bond energy (*E*_*HB*_) between the **C7** and water molecules was calculated using density functional theory simulations and was similar to that of hydrogen bonds in the bulk ice (Figure 2C3). The adhesion strength of **TC7**_10_-**H**_1_ was 4.15 MPa (tensile rate 1.38 MPa/s), which is significantly higher than that of commercial PVA adhesives (0.43 MPa).

Inspired by this work, Dong et al. extended the structural water system to a pillararene-crown ether (**PC**) system (Li et al., 2020b) in which ten **C7** units were decorated on the upper and lower rims of pillar[5]arene. The frequency-dependence of the BDS conductivity spectra for **PC**_10_-water (**W**)_1_ exhibited a similar discontinuity around the freezing point of water, indicating the existence of structural water. Intriguingly, the resulting CE-structural water supramolecular polymer system exhibited low-temperature-resistant adhesive properties. The resulting **PC**_10_-**W**_1_ complex exhibited adhesion strengths up to 2.49 MPa at −18°C. By contrast, the adhesion strengths of samples with a high water content were between 0.37 and 0.46 MPa, which is comparable to that of pure water (0.36 MPa). Notably, the structural water in the **PC**_10_-**W**_1_ sample remained liquid down to −80°C, indicating that structural water imparts anti-icing properties to the adhesive material. The macroscopic adhesive strength was maintained between 1.12 and 1.46 MPa at −80°C. By contrast, high-water-content **PC** samples became turbid solids at low temperatures because the free water in these samples froze. These studies elegantly demonstrate the potential of CEs with structural water as supramolecular adhesive materials (Zhang et al., 2019; Li et al., 2020c).

## Summaries and perspectives

This mini-review has highlighted selected, preeminent examples from the growing field of aqueous supramolecular chemistry of CEs. These studies have revealed new and unusual properties of CEs arising from their unique ability to interact with water molecules. These studies demonstrated the correlation between the hydration of CEs and the cyclic topological effect and the implications of cation affinities for neutral polar surfaces in the Hofmeister series. There is still much to explore and accomplish in this field; 1) CEs decorated with diverse functional groups make it easier to create diverse functional building blocks (Shen et al., 2019), thus enabling numerous opportunities for designing novel supramolecular materials in water; 2) To date, investigations into the aqueous behaviors of Se- or Te-containing CEs are scarce. The intrinsic catalytic roles and redox-responsiveness of Se and Te compounds significantly expand the functional range of CEs *via* substitution of a chalcogen atom on the CE with Se or Te (Shang et al., 2021a; Shang et al., 2021b; Li et al., 2022); 3) Revealing the interactions between water, CEs, and ions can benefit applied areas such as anti-icing materials (Ge et al., 2022; Hu et al., 2022) and aqueous metal-ion batteries (Wang et al., 2020). Aqueous CE supramolecular chemistry is an emerging research field and further studies will broaden our understanding of the properties and potential applications of aqueous CE systems.
